# Associations between self-care advice and healing time in patients with venous leg ulcer– a Swedish registry-based study

**DOI:** 10.1186/s12877-024-04660-8

**Published:** 2024-02-01

**Authors:** Marcus Rosenburg, Hanna Tuvesson, Gunilla Lindqvist, Lars Brudin, Cecilia Fagerström

**Affiliations:** 1 Faculty of Health and Life Sciences, Department of Health and Caring Sciences, Linnaeus University, Växjö, Sweden; 2 School of Health and Welfare, Department of Health and Nursing, Halmstad University, Halmstad, Sweden; 3 Department of Clinical Physiology, Kalmar County Hospital, Kalmar, Sweden; 4 Department of Health, Medicine and Caring Sciences, Linköping University, Linköping, Sweden; 5 Department of Research Region Kalmar County, Kalmar, Sweden

**Keywords:** Healing, Nutrition, Physical activity, Self-care advice, Venous leg ulcer

## Abstract

**Background:**

Venous leg ulcers take time to heal. It is advocated that physical activity plays a role in healing, and so does the patient’s nutritional status. Additionally, malnutrition influences the inflammatory processes, which extends the healing time. Therefore, the staff’s advising role is important for patient outcomes. Thus, this study aimed to investigate the associations between given self-care advice and healing time in patients with venous leg ulcers while controlling for demographic and ulcer-related factors.

**Methods:**

The sample consisted of patients registered in the Registry of Ulcer Treatment (RUT) which includes patient and ulcer-related and healing variables. The data was analyzed with descriptive statistics. Logistic regression models were performed to investigate the influence of self-care advice on healing time.

**Results:**

No associations between shorter healing time (less than 70 days) and the staff´s self-care advice on physical activity was identified, whilst pain (OR 1.90, CI 1.32–2.42, *p* < 0.001) and giving of nutrition advice (OR 1.55, CI 1.12–2.15, *p* = 0.009) showed an association with longer healing time.

**Conclusions:**

Neither self-care advice on nutrition and/or physical activity indicated to have a positive association with shorter healing time. However, information and counseling might not be enough. We emphasize the importance of continuously and systematically following up given advice throughout ulcer management, not only when having complicated ulcers.

## Introduction

Venous leg ulcers (VLUs) affect mainly older adults, incidence and prevalence seem to increase [1], which can be assumed to be associated with an increased number of older people. A recent study points out that there is inaccuracy in the prevalence and incidence of VLUs and a need for further studies. However, they report a pooled incidence of 0.17% and a pooled prevalence of 0.32% [2]. In Sweden, the prevalence of all leg ulcers is estimated at 0,52%, of which 36% are considered venous, meaning that about 20,000 individuals are affected by VLUs in Sweden [3]. VLUs derive from lesions in the venous circulation, where high BMI, low physical activity, and heredity are seen as contributing causes [4]. When an ulcer has occurred, pain, sleeping disturbance, and inhibited mobility affect the whole life [5]. Healing a VLU takes time; 50% takes more than a year to heal [6]. Prolonged healing affects the patient, who loses confidence in healing as well as the healthcare [7]. It has been reported that patients pend to contact the health care organization [8–10], which is assumed to risk a worsening of the patient’s quality of life and prolonging the healing time. Since VLUs commonly take time to heal, they have been referred to as chronic [11]. Later research rather discusses hard-to-heal ulcers [12–14], a description that is used by the Swedish National Registry for Ulcer Treatment (RUT) [15]. Regardless of whether VLUs are considered chronic or hard-to-heal, the risk factors and underlying causes of the ulcer bring a chronic increased risk for recurrence of new ulcers connected to new health care visits.

Malnutrition is frequently seen in patients with VLUs [16], and high BMI is common [17]. In some cases, increased body weight and muscle mass are desired. Then, prompt prescription of oral nutritional supplements, including calories and proteins, can facilitate healing [18, 19]. Furthermore, some patients ingest nutrients known to increase inflammation [17]. High BMI is also linked to a low activation rate, which, besides dietary changes, highlights the importance of physical activity. Furthermore, self-care measures such as compression stockings, weight loss, physical activity, smoking cessation, and elevating legs when/if possible are recommended [20]. Physical activity, involving resistance and/or foot/ankle is assumed to improve healing, with some evidence evolved recently [21]. A study focusing on reduced mobility and obesity concluded that it would be beneficial to acknowledge this impact to decrease the risk of developing VLUs [22]. An activation of the calf muscle through physical activity is recommended [23, 24]. However, the patients need information about their condition, the management, and advice regarding self-care [25], and what they can do by themselves, to reach a shorter healing time.

Ulcer management includes different pathways, starting with an adequate diagnosis and treatment, supplemented with self-care and prevention. The existing evidence for self-care in ulcer healing remains rather vague [26]. Additionally, much of the current research is outdated. Thus, the intention is to study the associations of self-care with ulcer healing. Patients with illness spend the majority of their daily lives without healthcare personnel. Thereby, some of the care, the self-care, falls on the patient [27], who should be acknowledged as important in the healing process. The World Health Organization uses a definition of self-care which reads: *“Self-care is the ability of individuals, families, and communities to promote health, prevent disease, maintain health and cope with illness and disability with or without the support of a health worker”* [28, 29]. In recent years, self-care has been highlighted as a measure for improving health and reducing the burden of symptoms in patients with VLU. Elevating legs, skin care, physical activity, and a healthy weight are mentioned [30] as examples of self-care activities. For the patient, self-care could include lifestyle changes, in the literature sometimes referred to as self-care maintenance. The overall aim of self-care is optimizing health and well-being, recognizing symptoms, and evaluating outcomes [31].

The healing time and the high rates of ulcer recurrence have the potential to decrease, but actions must be taken by the healthcare staff. Therefore patient education must be acknowledged as a crucial part of ulcer management as well as adherence to therapy [32, 33], and effective educational interventions have been requested [34, 35]. Several studies have shown that patients have not experienced satisfactory information during ulcer management, instead, the staff’s focus was on cleaning and dressing the ulcer [7, 36]. Trials with for example nurse-led interventions [37], explanatory images [38], or brochures [39] have shown positive results in terms of adherence and understanding. It is argued that the staff has a responsibility to assess and address the patient’s knowledge of ulcer management [40]. If behavioral changes are to occur, it is advocated that progress and goal fulfillment be continuously and carefully monitored and assessed [41]. A tailored communication for each patient is a foundation for achieving change and following the given advice [42]. Recently, healthcare staff in Sweden has a statutory duty to assess whether patients can perform self-care to prevent, examine, or treat diseases or injuries [43]. Efforts to advocate changes in diet and increase physical activity must be seen as an important part of ulcer management, however, more evidence is needed as the present findings of the impact of self-care advice are contradicting. Therefore, this study aims to investigate associations between given advice and healing time in ulcer management.

## Aim

This study aimed to investigate the associations between given self-care advice and healing time in patients with venous leg ulcers while controlling for demographic and ulcer-related factors.

## Methods

### Design

This study is a registry-based study using data from the Swedish National Registry of Ulcer Treatment (RUT) [44]. Descriptive statistics and logistic regressions were made to describe predictors (self-care advice) and their influence on the dependent variable, healing time.

### Settings

#### Swedish National Registry of Ulcer Treatment

The RUT, since starting in 2009 has more than 10 000 registrations of hard-to-heal ulcers. All ulcer types, that do not, or are not expected to heal within six weeks, are registered in the registry [15] of which venous ulcers are the most common etiology/diagnosis. In the registry, nurses, assistant nurses, and/or physicians report ulcers in a standardized and verified procedure [14]. Two registrations take place in RUT, at the first contact with the healthcare staff, when the diagnosis is set, and when the ulcer is healed (or when treatment is discontinued for some other reason) [45]. RUT consists of patients from different settings: primary care, hospital care, wound healing centers, community care, and private caregivers, with a national cover rate of 24% [46].

#### Study population

For this study, VLUs (*n* = 699), registered in RUT, between October 2015 and August 2020, were included. The VLUs included had all a follow-up date, and were healed. To be included in the present study the ulcers should be healed with a total duration of 6 weeks or more, which is in line with RUTs’ definition of a hard-to-heal ulcer [44]. Patients with a total ulcer duration of less than six weeks were excluded, so patients with ongoing ulcers, and patients with ulcers terminated for another reason (amputation, deceased, other) (*n* = 453). Additionally, 152 ulcers were excluded since only the first registered ulcer of each patient was included.

### Measurements

#### Background variables

At the first registration in RUT, information about sex (male/female), age (years), regular exercise (yes/no), BMI (body mass index) (range 14.2–65.7), and smoking habits were collected. Also, comorbidities; ongoing heart/lung/vascular disease (yes/no), ongoing diabetes (yes/no), ongoing rheumatoid arthritis/inflammatory disease (yes/no), ongoing neurological disease/paresis (yes/no) and ongoing malignant disease (yes/no) were registered. Each patient could be registered with none, single, or several morbidities.

#### Ulcer related variables

Different ulcer diagnosis was registered in RUT, but only VLUs were included in this study. Ulcer duration (before healthcare contact), registry date, and follow-up date were registered, and constitute fixed points of reference during ulcer management. Ulcer-related variables were also: ulcer-related pain (yes/no) and the prescribed ulcer treatment listed (compression therapy yes/no) as well as the number of staff monthly involved in the patient’s ulcer management (1->5) and the number of dressings every week (0->3).

#### Healing time

Healing time is reported in days and indicates the time from registration to healing. The variable was dichotomized, divided at the median i.e., less than 70 days and ≥ 70 days of healing time.

#### Self-care advice

Written advice which was given by the staff regarding nutrition (yes/no) and physical activity (yes/no) were registered in the RUT. This registration took place at follow-up and implies that the patient has received written advice sometime during ulcer management.

### Analysis

Data was analyzed with the IBM SPSS Statistics, version 28 and Statistica version 13.5.0.17 (TIBCO Software, Inc., Palo Alto, CA, USA). The characteristics of the sample were analyzed through descriptive statistics. The included sample was skewed, with some extremes in ulcer duration. This prompted to divide into two groups based on the median of ulcer duration (< and ≥ 84 days) (Table 1). Differences between the two groups of ulcer duration were analyzed using Mann-Whitney’s U-test for continuous and Chi-2 for categorical variables. A correlation matrix was set up, presenting correlations between all variables in the analysis. The relationships in the matrix were tested with Spearman´s rank correlation coefficient. To show distribution, in age, duration of treatment, and BMI, quartiles are reported.

**Table 1:** The study population’s demographics and clinical characteristics at registration calculated according to venous ulcer duration before registration

			Duration before registration					
Variable			<=84 days		> 84 days		p*	Total
N			358		341			699
Sex (%)								
	Male		145 (41)		114 (33)			259 (37)
	Female		213 (59)		227 (67)		0.053	440 (63)
Age (y)								
	Median (quartile range)		76 (69–84)		78 (71–85)		0.056	77 (70–85)
Age quartiles, n (%)								
	≤ 69		90 (25.1)		73 (21.4)			163 (23.3)
	69–77		98 (27.4)		81 (23.8)			179 (25.6)
	77>-84		85 (23.7)		82 (24.0)			167 (23.9)
	> 84		85 (24)		105 (31)		0.162	190 (27)
Body mass index (BMI; kg/m^2^)								
	Median (quartile range)		27 (24–32)		27 (24–31)		0.926	27 (24–31)
Body mass index (BMI; kg/m^2^)								
	≤ 24		65 (18.2)		67 (19.6)			132 (18.9)
	24>-27		57 (15.9)		65 (19.1)			122 (17.5)
	27>-31		66 (18.4)		72 (21.1)			138 (19.7)
	> 31		68 (19.0)		72 (21.1)		0.955	140 (20.0)
	Missing		102 (28.5)		65 (19.1)			167 (23.9)
Smoking, n (%)								
	Never smokers		221 (61.7)		225 (66.0)			446 (63.8)
	Ex-smoker		83 (23)		78 (23)			161 (23)
	Smokers		38 (10.6)		31 (9.1)		0.668	69 (9.9)
	Missing		16 (4.5)		7 (2.1)			23 (3.3)
Duration of treatment from registration								
	Median (quartile range)		60 (35–106)		84 (42–181)		< 0.001	70 (36–140)
Duration of treatment from registration								
	≤ 70 days		202 (56.4)		148 (43.4)			350 (50.1)
	> 70 days		156 (43.6)		193 (56.6)		< 0.001	349 (49.9)
Regular exercise			221 (61.7)		225 (66.0)		0.960	446 (63.8)
Pain (yes)			198 (55.3)		190 (55.7)		0.976	388 (55.5)
Comorbidity, n (%)								
	Diabetes mellitus		74 (20.7)		63 (18.5)		-	137 (19.6)
	Heart/Lung/Vascular		245 (68.4)		252 (73.9)		-	497 (71.1)
	Inflammatory		37 (10.3)		43 (12.6)		-	80 (11.4)
	Neurological		20 (5.6)		24 (7.0)		-	44 (6.3)
	Malignancies		21 (5.9)		12 (3.5)		-	33 (4.7)
Number of comorbidities n (%)								
	0		84 (23.5)		69 (20.2)			153 (21.9)
	1		184 (51.4)		174 (51.0)			358 (51.2)
	2		79 (22.1)		86 (25.2)			165 (23.6)
	3		11 (3.1)		12 (3.5)		0.642	23 (3.3)
Compression therapy								
	No		16 (4.5)		16 (4.7)			32 (4.6)
	Yes		340 (95.0)		320 (93.8)		0.989	660 (94.4)
	Missing		2 (0.6)		5 (1.5)		-	7 (1.0)
Staff per month								
	1–2		260 (72.6)		186 (54.5)			446 (63.8)
	3–4		59 (16.5)		69 (20.2)			128 (18.3)
	≥5 per month		9 (2.5)		15 (4.4)		0.011a	24 (3.4)
	Missing		30 (8.4)		71 (20.8)			101 (14.4)
Dressings per week (mean)								
	0-1.5		136 (38.0)		92 (27.0)			228 (32.6)
	1.5-2		215 (60.1)		233 (68.3)			448 (64.1)
	≥3		7 (2.0)		16 (4.7)		0.002b	23 (3.3)
Given self-care advice								
	Nutrition		175 (48.9)		163 (47.8)		0.836	338 (48.4)
	Physical activity		212 (59.2)		177 (51.9)		0.186	389 (55.7)
	Combination nutrition; activity		162 (45.3)		147 (43.1)		0.966	309 (44.2)

Footnotes to the table: (a) The only significant difference was between 1–2 and 3–4 (*p* = 0.019). (b) The only significant difference was between 0-1.5 and 1.5-2 (*p* = 0.005)

Given advice of self-care, demographic and ulcer-related variables were entered in univariate logistic regression models to investigate variables significantly associated with the outcome variable healing time less than 70 days or more than 70 days. A *p*-value of less than 0.05 was considered to indicate statistical significance. Variables significantly associated with healing time in the univariate logistic regression models together with age and sex were then entered into a multivariate logistic regression analysis, using backward stepwise selection. The multivariate logistic regression was here performed to express the independent contribution of the included potential determinants (self-care advice of nutrition and physical activity) to healing time when controlling for ulcer-related variables adjusted for sex and age. The results from the logistic regressions are presented with Odds Ratios (OR) with 95% confidence intervals (CI). Odds ratios were used to indicate associations between variables [47].

### Ethics approval and consent to participate

The Swedish Ethical Review Authority has approved the realization of the present study (nr 2020 − 00965), according to the Swedish law on research [48]. The approval enabled data extraction from the quality registry. Data without social security numbers or names was retrieved from the registry after approval by the Uppsala Clinical Research Center. The Helsinki Declaration was a guideline throughout the work of this study, among other things regarding the storage of patient data and confidentiality [49]. In connection with registration, written consent was obtained regarding storage of data in the RUT, and the use of data in quality development and research. Registration in RUT is governed by Swedish law, including the Publicity and Privacy Act (2009:400) [50], the Swedish Act with supplementary provisions to the EU’s Data Protection Regulation (GDPR) (2018:218) [51], and the Patient Data Act, Chap. 7 (2008:355) [52].

## Results

The sample consisted of *n* = 699 patients with VLUs. The median age was 77 years and 63% were female. Low BMI (< 24) is seen in 19% of the sample, whereas 40% have a BMI higher than 27. In the sample, 10% were smokers. Pain is reported in 56% of the patients. More than 25% of the sample had two comorbidities or more. Heart/vascular/pulmonary disease was the most common comorbidity in the sample, followed by diabetes. In the sample, 660 patients had compression as part of ulcer management.

As seen in Table 1, there is a relationship between having a longer duration (≥ 84 days) before the first encounter, and a longer period of treatment, more dressing per week (> 3 times), and the number of staff involved in the management (≥ 5 per month) (*p* ≤ 0.011), compared to having a shorter duration before the first encounter. Within the sample, 64% were conducting regular exercise. Most of the patients, 64%, were managed by 1–2 staff per month and 64% had their dressings changed on average 1.5-2 times per week.

In VLUs with a duration of ≥ 84 days before registration, 52% of the 341 patients got self-care advice regarding physical activity. The results show that it is common for patients to have the ulcer rather long before seeking healthcare; almost every second patient had an ulcer 84 days or more before the first encounter. Self-care advice on nutrition was given in 48% of the patients, and on physical activity in 56% respectively. Advice on both nutrition and physical activity was given in 44% of the sample.

In the sample included in the logistic regression models, 49% of the females and 52% of the males had a healing time of 70 days or longer. As seen in Table 2, 54% of those with a BMI < 24, had healing longer than 70 days. In the group of patients with a BMI over 31, 53% had a healing time longer than 70 days. Among those with a longer healing time, regular exercise at first registration was slightly less frequent (49%). In the univariate regression models, longer duration of ulcer before registration entailed a significant (*p* < 0.001) longer healing time (> 168 days: OR = 2.09, CI = 1.41–3.07), (84–168 days: OR = 1.62, CI = 1.26–2.11), (56–84 days: OR = 1.28, CI = 1.12–1.45). Furthermore, pain (OR = 1.79, CI = 1.32–2.44, *p* < 0.001) entailed longer healing time (more than 70 days). The univariate logistic regression also showed an association between longer healing time and a high number of staff involved per month (> 4) (OR = 2.13, CI = 1.16–3.92, *p =* 0.015), the number of ulcer dressings per week, ≥ 3 (OR = 1.75, CI = 0.99–3.11, *p* = 0.054) and nutritional advises (OR = 1.42, CI = 1.04–1.95, *p* = 0.028) whilst advice on physical activity was not significant for healing time (Table 2).

**Table 2:** Odds ratios (OR) for longer healing vs. shorter healing of venous leg ulcers were analyzed using logistic regression. Univariate to the left and multivariate (adjusted for age and sex) to the right

			Healing > 70 days			Univariate logistic regression			Multivariate logistic regression	
Variable		Total	n	(%)		OR (95% CI)	*p*		OR (95% CI)	*p*
Sex										
	Female	440	214	49		1.00			1.00	
	Male	259	135	52		1.15 (0.85–1.56)	0.373		1.11 (0.79–1.57)	0.553
Age categories										
	<=70	179	87	49		1.00			1.00	
	69–77	163	81	50		1.02 (0.89–1.17)			1.04 (0.90–1.21)	
	77>-85	192	100	52		1.04 (0.79–1.36)			1.09 (0.81–1.47)	
	> 85	358	176	49		1.05 (0.70–1.59)	0.804		1.14 (0.73–1.79)	0.573
Body mass index (kg/m^2^)										
	<=24	132	71	54		1.00			-	
	24>-27	122	55	45		1.01 (0.87–1.18)			-	
	27>-31	138	70	51		1.02 (0.75–1.39)			-	
	> 31	140	74	53		1.03 (0.65–1.65)	0.890		-	
	Missing	167	79	47						
Number of comorbidities										-
	0	153	76	50		1.00			-	
	1	358	176	49		1.05 (0.86–1.28)			-	-
	2	165	84	51		1.10 (0.75–1.64)			-	-
	3	23	13	57		1.16 (0.64–2.09)	0.619		-	
Duration before registration (days)										
	<=56	237	106	45		1.00			1.00	-
	56>-84	121	50	41		1.28 (1.12–1.45)			1.32 (1.14–1.52)	
	84>-168	184	92	50		1.63 (1.26–2.11)			1.74 (1.31–2.31)	
	> 168	157	101	64		2.09 (1.41–3.07)	< 0.001		2.29 (1.50–3.50)	< 0.001
Regular exercise										
	No	373	190	51		1.00			-	
	Yes	277	135	49		0.92 (0.67–1.25)	0.581		-	
	Missing	49	24	49					-	
Pain										
	No pain	295	123	42		1.00			1.00	
	Pain	388	218	56		1.79 (1.32–2.44)	< 0.001		1.90 (1.36–2.64)	< 0.001
	Missing	16	8	50						
Staff per month										
	1–2	446	203	46		1.00			-	
	3–4	128	64	50		1.46 (1.08–1.98)			-	
	> 4	24	18	75		2.13 (1.16–3.92)	0.015		-	
	Missing	101	64	63					-	
Dressings per week (mean)										
	0-1.5	228	103	45		1.00			-	
	1.5-2	448	232	52		1.32 (0.99–1.76)			-	
	≥ 3	23	14	61		1.75 (0.99–3.11)	0.054		-	
Compression therapy										
	No	32	20	63		1.00			-	
	Yes	660	323	49		0.58 (0.28–1.20)	0.139		-	
	Missing	7	6	86		-				
Given self-care advice on nutrition										
	No	291	131	45		1.00			1.00	
	Yes	338	182	54		1.42 (1.04–1.95)	0.028		1.55 (1.12–2.15)	0.009
	Missing	70	36	51					-	
Given self-care advice on physical activity										
	No	242	116	48		1.00			-	
	Yes	389	195	50		1.09 (0.79–1.51)	0.597		-	
	Missing	68	38	56					-	

The reference value for healing time is < 70 days

In the multivariate logistic regression analysis, the importance of longer duration before registration shows a significant trend, with an average OR at 1.32 between groups. Even in the multivariate logistic regression, the presence of pain (OR = 1.90, CI = 1.36–2.64, *p* < 0.001 were associated with longer healing time (> 70 days). In addition, self-care advice on nutrition (OR = 1.55, 95% CI = 1.12–2.15, *p* = 0.009) showed an impact on longer healing time (> 70 days).

## Discussion

Due to the chronicity in the underlying causes of the ulcer, and the high rates of recurrence [53], a lasting change of lifestyle for shortened healing time, ulcer management, and ways of reducing the risk for recurrence are considerable [26]. Previously, it has been reported that patient knowledge regarding ulcer genesis and ulcer recurrence is insufficient [34, 35, 54], which increases the risk of a prolonged healing process due to lack of adherence to the treatment plan and given advice. Nurses and other healthcare staff play an essential role in advising patients on self-care, and thorough, and clear information is considered crucial [39, 55]. This study did not find any associations between advising on self-care and shorter healing time, however, there are associations between not having pain and shorter duration before registration with shorter healing time.

The result from the present study shows that more than every second patient experienced pain (55%) which also was a condition that had a significant impact (OR 1.90) on ulcer healing time. I.e., patients who reported pain are 90% more likely to have longer healing time, more than 70 days, when adjusting for age and gender. The prevalence of pain when having VLU is in line with the results from Wickström et al. (2020), who investigated data from the same registry, but with a focus on pain amongst all patients registered, not only VLUs. In their study, the participants, of which 44% had venous ulcers, reported a mean VAS score of 5.7 (SD 2.3) (range 1–10) [13]. Another study on pain in VLUs acknowledges pain to be a significant problem, in that study 85% of patients reported pain [56].

This study could not detect positive associations between self-care advice and shorter ulcer healing time. The multivariate logistic regression analysis revealed that the odds ratio for longer healing time was 55% higher in the patients given self-care advice on nutrition. This reverse association is an unexpected finding, as previous studies [18, 19] have indicated that positive relationships between shorter healing and nutrition have been observed. The result might be explained by that information about self-care advice on nutrition and dietary supplements are more often included as an element in ulcers of greater severity when the ulcer takes a longer time to heal. It is known that ulcer management influences the staff, sometimes through frustration over the healing process [57]. Irrespective of underlying causes of the probability of giving advice, only 338 (48%) patients got this advice, despite the nutrition’s undoubted importance for healing. Some patients need advice to gain weight, while others need to lose weight [15]. A profound focus on nutrition has previously shown positive results on healing [15, 17]. Several studies stress that nutrition can have positive effects [16, 17, 48, 49]. Furthermore, it is known that some nutrients are associated with inflammation, and some nutrients are required for healing [17–19]. Thereby, an increased focus on possible gains of nutrition and physical activity in ulcer management is emphasized.

Interesting to note is that just every second (56%) of the sample received self-care advice on physical activity at the same time, it seems that this measure has no significant impact on healing time. Nevertheless, physical activity can be beneficial in terms of the influence of weight loss which can have a positive relation to ulceration [58] since patients with obesity are at higher risk for VLUs [22]. Furthermore, an activation of calf muscles facilitates the venous reflux [23, 24]. Also, the overall health is favored by physical activity [59] besides, the impaired ability risks worsening other concurrent conditions [60].

In summary, giving advice appears to not be enough when it comes to achieving healing. The staff’s role in supporting patients in self-care activities and giving advice is described in the literature. Continuous work with support, information, and behaviors has the potential to increase confidence in and the use of self-care [61]. To perform self-care requires an awareness, resurrected, among other things, from knowledge [27]. Also within empowerment, knowledge is acknowledged, and patients understanding their illness contributes to control [62]. Here, staff should emphasize a clear, targeted, and personalized focus when sharing information and advising VLU patients. One possible way to go is through person-centering, a recognized measure to collect data from the patient and customize information [42, 63]. The relationship with the healthcare staff is experienced as crucial and has been reported to be inferior [7, 64, 65]. In the present study, the median healing time was 70 days, and most of the patients had 1.5-2 dressings per week. A long time of ulcer management provides possibilities to build a genuine relationship with the patient with VLU.

There is a need for clear methods for advising patients with VLUs at an early point in VLU management to be able to influence and shorten the healing process. Ulcer management is commonly time-consuming, which is an opportunity to support the patient with an upgraded lifestyle regarding nutrition and physical activity. As mentioned, 50% of patients are not fully advised, which means there is an important job ahead to meet the need for advising all patients with VLUs, especially as it is included in several guidelines [25, 66–69].

### Clinical relevance of the results

Pain is associated with longer healing time. Regardless of whether pain occurs in the most hard-to-heal ulcers or if the most painful ulcers are harder to heal– pain is common and must be addressed. Lifestyle advice is a recognized measure in the management of VLU. This study did not stress an association between advice and shorter healing time. Therefore, further research is needed to clarify the structure and content of the information given. With a shorter healing time as a target, improved patient involvement, and valorization of her/his resources in ulcer management need to be recognized. Notwithstanding that evidence for nutrition and physical activity in terms of the healing time is still rather vague, and more evidence on the value of systematic and inclusive patient involvement in ulcer management is emphasized.

### Limitations

The present study is based on VLUs registered in the RUT, a Swedish national registry, with verified variables. It is a strength that ulcers from different settings are included since patients are managed throughout Sweden’s whole healthcare system. The great number of patients in the registry entails a certain generalizability to patients with VLUs [70]. Each ulcer is registered, and most of the variables are completed for each ulcer, which allows us to say results of the present study are reliable. However, including recently registered patients, might change the results, as ulcer management could have evolved. This study represents VLUs registered after October 2015 and healed before the end of August 2020. We decided to exclude ulcers with a total duration of less than 6 weeks, which is in line with the use of the term hard-to-heal ulcers. Furthermore, patients’ successive ulcers were excluded and since registration of given advice is registered in connection with registration of healing, only healed ulcers were included. Including only healed ulcers entails that patients that did not heal, may nevertheless have received self-care advice.

The fact that the use of RUT is optional, must be addressed. One-fourth of units in Sweden treating ulcers enter data into the registry. Thus, there might be a risk that only the most engaged units with staff and leadership that see the importance of structured management are included. However, as we provide data regarding the patients in the RUT, it could be assumable that patients not registered receive even less coherent and structured ulcer management. Previous studies have highlighted quality registries as an important source of data, for research and for assessment of provided healthcare [71–73].

The same for every registered patient is that they received written information during ulcer management, without any specified time. As a “no” means that no advice is given, and any reason is not mentioned, meaning that an assessment of the patient’s prior knowledge could have been done, confirming a great understanding of its message. A possible limitation of this study is that the information may differ between regions, as well as between units. Nationally there is no uniform directive available on how and what information patients with VLU should receive, which may create uncertainty in ulcer management and a deficiency in the matter of being able to maintain quality and patient safety. However, the current way to inform patients leaves an opportunity to prioritize information according to the patient’s needs in ulcer management.

Another feasible limitation is that data from the register have no information about the exact level of compression in mmHg only what type of compression treatment they have registered (i.e. sock class, short stretch, middle elastic, multilayer, and tube bandages). Since compression is renowned for its role in accelerated ulcer healing [74, 75] we still chose to go further and use a dichotomous variable of compression (yes/no) in the analysis and the univariate logistic regression. Since its importance was not significant, it was not part of the multivariate logistic regression.

In addition to ulcer duration, wound size is a central variable for healing time [76]. In the dataset from the RUT register, size was only reported in 25% of the cases and the inclusion of a variable with such a large dropout would affect the number of cases included in the regression model with the risk of losing information. Measuring ulcers differs depending on whether it is carried out manually or digitally and the authors in a previous study considered that ulcer measuring was difficult to use and control which means limitation [77]. The data collection for this study was in 2015–2020 and the technology for measuring ulcers has improved since then, especially in recent years, and shows increased evidence of its certainty [78].

## Conclusions

The present study could not reveal a significantly shorter healing time when advised on nutrition and/or physical activity in patients with VLUs. Despite good intentions when advising patients in self-care activities such as physical activity and nutrition, advising per se does not mean that the patient acts on it. On the other hand, pain and ulcer duration before registration is associated with longer healing time. The patient with VLU is often an older person, with one or more comorbidities, a fact that must be recognized. This study could reveal that about every second patient was advised on self-care activities, something that is considered an important part of ulcer management. We emphasize the need for a continuous focus on the structure and content of information, follow-up of understanding, and cooperation with the patient in ulcer management.

## Data Availability

Due to an agreement with the provider of the data from the quality registry, data is not shared. However, any questions about the data are referred to the corresponding author.

## Abbreviations

RUT: (Swedish) Registry of Ulcer Treatment
VLU: venous leg ulcer
